# Prevalence and demographic risk factors for overweight and obesity among healthcare workers at Uasin Gishu County hospital, Kenya

**DOI:** 10.4314/ahs.v23i2.65

**Published:** 2023-06

**Authors:** Rael Sum Jepchumba, Ann Munyaka, Regina Kamuhu

**Affiliations:** Kenyatta University, Food Nutrition and Dietetics

**Keywords:** Prevalence, BMI, overweight, obesity, demographic risk factors, healthcare workers

## Abstract

**Background:**

Healthcare workers are under continual pressure to be an example to the public on Body Mass Index (BMI). While prior studies have focused on the general population, data on overweight and obesity is limited among healthcare workers.

**Objective:**

This study aimed at determining the prevalence of and demographic risk factors associated with overweight and obesity among healthcare workers.

**Methods:**

This cross-sectional study was done on healthcare workers aged 20 years and above comprehensively sampled in Uasin Gishu County hospital. A WHO step-wise questionnaire and anthropometric measurements were used to collect data and logistic regression was performed among variables.

**Results:**

The mean age and BMI were 36.96±9.96 years and 27.18±5.04 respectively. 63.4% of respondents had their BMI above 25 kg/m^2^, 35% were overweight and 28.4% were obese. Females were more likely to become overweight and obese than males with an odds ratio [OR] of 2.8 (95% confidence interval [Cl] = 1.3-6.0, P = 0.008). Age, education and physical activity were found related to BMI and gender was found to be associated with BMI.

**Conclusion:**

High prevalence of obesity among healthcare workers is of concern, especially on the issue of central obesity. Intervention among the health workers should be considered.

## Introduction

Overweight and obesity are increasing globally and are described as conditions of the 21^st^ century that needs attention. These conditions result from excess energy intake that accumulates as fat in the body. Overweight and Obesity are major risk factors for disease and death. Overweight (BMI ≥25 to ≤29.9 kg/m2) and obesity (BMI ≥30 kg/m^2^) are associated with mortality and morbidity especially from cardiovascular diseases such as hypertension, stroke, ischemic heart diseases and diabetes 1. This phenomenon is becoming a public health concern in both developing and developed countries. Obesity is contributed by many factors including food choices such as consumption of fast foods due to urbanization with improved economic status and reduced physical activity levels among others 2-4.

According to the WHO report published in 2016, the global prevalence of obesity and overweight was 39% in adults aged 18 years and above. More than 1.9 billion adults aged 18 years and older were overweight and over 650 million of these adults were obese^5^. The prevalence of overweight and Obesity in different regions are 62.5% in America, 58.7% in Europe, 31.7% in Western pacific with the lowest prevalence being observed in South East Asia at 21.9% 6,7. Prevalence of obesity in Kenya has more than doubled from 6.4% in 1993 to 15% in 2014 8. Data from the Kenya Demographic Health Survey 2009^9^ gives the prevalence of both overweight and obesity at 23%. National prevalence of obesity and overweight among women of reproductive age in Kenya rose from 25% in 2008 to 33% in 2014 10. Studies show an increase in the prevalence of overweight and obesity in women than in men 2,11-13.

Among the groups that have high risks of obesity and overweight in the general population are healthcare workers 14. Compelling demands at job such as night shifts, work stress among doctors and nurses and workload are some of the risk factors of overweight and obesity among healthcare workers 15. Prevalence of overweight and obesity of 58.8% among healthcare workers in Kisumu east sub-county, Kenya was previously reported 14. The cause of this high prevalence of overweight and obesity among Health workers is not yet clearly understood. Due to lack of up-to-date and specific data on the prevalence of overweight and obesity among healthcare workers, this study seeks to determine the prevalence of overweight and obesity and demographic risk factors among health care workers at Uasin Gishu County Hospital, Kenya.

## Methods

A cross-sectional analytical study design was adopted. Comprehensive sample size was used to sample healthcare workers at Uasin Gishu County Hospital which was purposively sampled since it is the largest county hospital. The hospital had one hundred and sixty-nine workers and all of them were qualified for the study. However, after excluding those who were sick, pregnant and away on leave at the study time, 136 healthcare workers both males and females participated in the study who were conveniently sampled. Socio-demographics, alcohol use and anthropometric data were collected by the use of a self-administered questionnaire, which was modified after pre-testing from a WHO stepwise-structured questionnaire. The questionnaire was pretested on 14 hospital staff at Moi Teaching and Referral Hospital and adjustments made according to feedback to ensure reliability and clarity. Calibration of instruments was done before the research for accuracy.

### Data Collection

A WHO stepwise-structured questionnaire was used to collect data by way of face-to-face interviews then followed by anthropometric measurements done by healthcare worker. The health service worker was trained on how to carry out interviews and anthropometric protocols. Data collection process was done within the hospital every day for two weeks until the target population was met.

### Anthropometric Measurements

Anthropometric measurements were taken using a digital Seca® 813 scale for weight with measurements to the nearest 0.1kg with the respondent having the least cloth and standing barefooted. Height was taken using a Seca® 213 stadiometer and recorded to the nearest 0.1cm with the participant standing erect and their back and buttocks touching the wall and their feet on flat ground. Waist and hip ratio (WHR), an indicator for central obesity, was determined by measuring waist circumference and hip circumference using a 203 cm Seca® measuring tape. Waist circumference measurements were taken at midpoint between the iliac crest and lower border of the tenth rib while hip circumference measurements were taken around the widest portion of the buttocks with the tape parallel to the floor. Participants were standing with feet closed together, arms at the side with bodyweight evenly distributed and with little clothes for both measurements which were done twice and an average to the nearest 0.1cm computed.

### Statistical analysis

Data was cleaned, coded and entered to the Statistical Package for Social Sciences software (SPSS). Descriptive statistics such as means; standard deviation was used for continuous data. BMI was calculated as weight in kilogram divided by the square of height in meter (kg/m^2^) and classified as per the WHO classification of obesity (BMI>30 kg/m^2^), overweight (BMI 25-29.5 kg/m^2^), normal BMI (18.5-24.9 kg/m^2^) and underweight (BMI<18.5 kg/m^2^). Waist hip ratio was calculated by dividing waist circumference by hip circumference measurements and WHR of > 0.85 in female and >0.90 in males were taken as central obesity as categorized by WHO cut off points. Inadequate physical activity was classified as less than 600 metabolic equivalents (METs) in a week while adequate amount of physical activity above 600 METs in a week. Descriptive statistics was done and results presented by frequencies and percentages with mean and standard deviations. Chi-square and odds ratio was used to determine associations between categorical data and logistic regression was done for relationships among variables. A p-value of less than 0.05 at 95% confidence interval was considered to indicate statistical significance.

### Ethical approval

Study was conducted with an approval of Kenyatta University Ethical Review Committee with a reference number: KU/ERC/APPROVAL/VOL.1(154). A research permit was obtained from the National Council of Science, Technology and Innovations (NACOSTI). Written permission was sought from county health administrator Uasin Gishu County and medical superintendent of Uasin Gishu County Hospital before commencing the research work. Verbal and written informed consent were obtained from respondents before data was collected.

## Results

### Socio-demographic characteristics of the study participants

A total of 136 healthcare workers were interviewed. The respondents interviewed comprised of 51 (37.5%) males and 85 (62.5%) females. Mean age was 36.96±9.96 years within the range of 21-58 years. The age group 3140 had the majority of the respondents. More than half (69.9%) of the study participants were married. Almost all (95.6%) of the subjects had tertiary education. About thirty per cent of the participants were earning between Ksh 30000-50000, 27.9% were those earning less than KSH 30000. Staff in the clinical cadre were 52% of participants while the rest were from non-clinical cadre (Table 1).

**Table 1:** Socio-demographic and economic characteristics of healthcare workers of Uasin Gishu County Hospital

Age in years	Male n=51 (%)	Female n=85 (%)	Totals n=136 (%)
**21-30**	33.3	29.4	30.9
**31-40**	31.4	32.9	32.4
**41-50**	29.4	24.7	26.5
**51 & above**	5.9	12.9	10.3

**Variable**	**Category**	**Percentage**	χ^2^ p-value

**Marital status**	Never married	19.9	0.513
	Currently married	69.9	
	Separated	0.7	
	Divorced	2.2	
	Windowed	2.9	
	Cohabiting	4.4	

**Education**	Secondary,	4.4	
	College/University	95.6	0.012*

**Income**	Less than 30000	27.9	
	30001-50000	29.5	
	50001-70000	17.6	
	70001-90000	16.2	
	90001-110000	5.9	

### Prevalence of overweight and obesity

**Figure 1:**
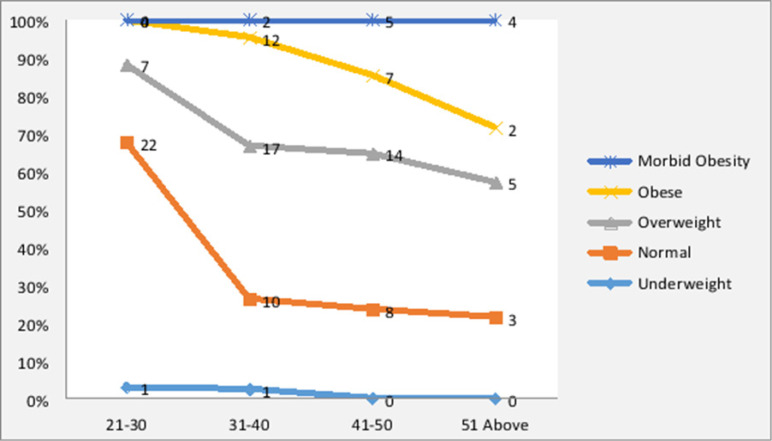
Distribution of frequency of BMI according to Age groups of Uasin Gishu County Hospital Health workers

The overall prevalence of obesity and overweight was 63.4%. About 1.6% of respondents were underweight a BMI<18.5 kg/m2, 35% had normal weight, while 35% and 28.4% were overweight and obese respectively. The mean BMI of respondents was 27.18±5.04. More female respondents (27.0%) were found to be obese as compared to male counterparts (10.2%) and were found to have inadequate physical activity of 22% (Table 2). More females than males had morbid obesity. Central obesity was present in the majority of the study participants and was prominent in the age groups 31-40 and 41-50 (Figure 1). The mean waist-hip ratio was 0.8811±0.8328 in female respondents and 0.9213±0.8023 in male respondents indicating central obesity.

**Table 2:** Nutritional status of Uasin Gishu County Hospital Healthcare workers based on BMI and WHR Categories

Category	BMI	χ^2^ P-value	0.007				WHR	0.002

	Underweight	Normal	Overweight	Obese		Morbid obese	Normal	Central obesity
**Totals (%)**	1.6	35	35	20.3		8.1	33.1	66.9
**Male (%)**	4.1	46.9	36.7	10.2		2.0	31.2	68.8
**Female (%)**	0.0	27.0	33.8	27.0		12.2	34.2	65.8

**Category**	**Met Equivalent**							

	**Inadequate**	**(<600MET)**	**Adequate**	**(>600MET)**				

	**Frequency**	**Proportion**	**Frequency**	**Proportion**	χ^2^ P-value			

Male	0.0	0.0	5 1	100.0	0.002*			
Female	19	22.5	66	77.5	0.001*			
Total	19	14	117	86.0				

In regard to the distribution of BMI according to age, advanced age group (41–50-year-olds) showed the highest number of people who were obese than the other age groups (Figure 1).

### Predictors of overweight and obesity

On analysis logistic regression test revealed that age was related with BMI and this indicates that age is one of the risk factors for obesity. It was also noted that physical activity was negatively related with BMI (Table 3). Education, household number and alcohol use were also found to significantly relate to BMI. This are among the many predictors of overweight and obesity among the health workers. Chi-square test revealed that gender was associated with BMI (p-value 0.007) among respondents. Besides, females were significantly more likely to become overweight and obese than males with an OR of 2.8 (95% Cl = 1.3-6.0, P = 0.008).

**Table 3:** Relationships between selected variables of Uasin Gishu Hospital Staff

Relationships	R	p-value
Age and BMI	0.348**	<.001
Income and BMI	0.099	0.276
Education and BMI	0.261*	0.016
HH number and BMI	0.258**	0.004
Income and BMI	0.225*	0.012
Alcohol use and BMI	0.220*	0.015
Physical activity and BMI	-0.100	0.271

* Correlation is significant at the 0.05 level (2 tailed).

** Correlation is significant at the 0.01 level (2-tailed).

## Discussion

In the current study, the overall prevalence of overweight and obesity was 35% and 28.4%, respectively. A BMI of more than 25kg/m2 and central obesity is associated with >200 comorbidities related to morbidity and mortality 16. For instance, overweight and obesity are important concerns for healthcare workers since they predispose them to non-communicable diseases such as hypertension, diabetes, cancers and cardiovascular diseases. The aforementioned conditions will lower the productivity of the health workers and these will affect the services rendered by this group. In our study, more than half of the respondents (63.4%) were found to be either overweight or obese. This finding is consistent with that reported in a study conducted in 2012 showing that the prevalence of overweight and obesity among Brazilian health care professional to be 66% 11. An increase in prevalence of overweight and obesity among health workers was observed in Kenya at 58.8% in Kisumu east sub county in 2016 17. High prevalence of obesity and overweight can be attributed to so many factors one being food transition from the traditional high fibre foods to fast foods which are highly refined with a lot of fat, sugar and salt. This transition always comes with several factors such as stable income, changes in the food industry on food production and lack of better food choices in the market forcing people to settle for the available ones that are not healthy 3.

Health care practitioners are among the many people affected by overweight and obesity since they work in shifts and sometimes long hours which make them inclined towards fast foods available in the market due to time constraints. The prevalence of obesity was found to be higher in female respondents than male respondents and Chi-square analysis revealed a significant association between gender and BMI. The higher prevalence of obesity observed in the current study among female respondents compared to male respondents is in agreement with reports of previous studies 17-18. Age, economic status, physical activity level, education and household number are the main predictors of overweight and obesity in this study and in agreement with other studies among women in Nairobi County 6,17. In other studies, it has been shown that women gain the greatest amount of weight in their childbearing age and a majority do not lose this gestational weight gained, therefore, become overweight and obese 6.

In the current study, 35% of respondents were found to be overweight and this is close to value of 38.4% reported in 2018 among health care workers in Ghana 18. Another study carried out by Mitwalli 15 among health professionals in Saudi Arabia reported that 19.4% of them were obese. Central obesity which is strongly associated with cardiovascular diseases was more prevalent among the current study participants. Obesity is associated with an increased rate of absenteeism, morbidity, mortality, work-related injuries, decreased productivity and early retirement which in turn increase DALY 20. Moreover, central obesity is linked to cardiovascular diseases and other non-communicable diseases such as diabetes and cancers. Furthermore, health practitioners are to be an example of body weight as well as educators and health promoters^21^. They need to be an agent of change in the fight against overweight and obesity by appearance than theoretical education 5,19,21.

## Limitation of the study

This study had several limitations. First, we did not report confounding risk factors such as dietary practices. Also, we did not examine the correlation between anthropometric measures and metabolic profile which can be a further disease risk factor. Since this study was a cross-sectional, casual relationships between risk factors and BMI were not established.

## Conclusion

There is an increase in obesity and overweight among health care workers especially among females and those with advanced age. Demographic risk factors among healthcare workers emerge to be the predisposing risk factors to overweight and obesity. This condition predisposes them to raised blood sugar and high blood pressure. However high socio-economic status, physical inactivity, education and household number contributed to obesity among the healthcare workers. This contributes to disability adjusted life years and reduce effectiveness of an individual.

## Recommendations

Policy makers such as ministry of health and other relevant stakeholders should consider categorizing policies to specific groups like healthcare workers to help them have appropriate lifestyle at work that promote a healthy weight. More research should be done to establish the emerging risk factors of overweight and obesity and evidenced informed interventions be put in place. Promote healthy eating habits by providing nutritious and healthy foods that are low in fats, sugars and salt with traditional vegetables and whole grains in food joints at the health facilities that are affordable. Periodical screening of their nutritional status should be done. Individualized and Taylor made planned intervention should be given to members. Promote strategies among health care workers to increase on physical activity.
